# The Complex Plank Problem, Revisited

**DOI:** 10.1007/s00454-022-00423-7

**Published:** 2022-08-27

**Authors:** Oscar Ortega-Moreno

**Affiliations:** Vienna University of Technology, Vienna, Austria

**Keywords:** Complex geometry, Discrete geometry, Functional analysis, Covering problems, Plank problems, 52C99, 51M16, 46C05

## Abstract

Ball’s complex plank theorem states that if $v_1,\dots ,v_n$ are unit vectors in ${\mathbb {C}}^d$, and $t_1,\dots ,t_n$ are non-negative numbers satisfying $\sum _{k=1}^nt_k^2 = 1$, then there exists a unit vector *v* in ${\mathbb {C}}^d$ for which $|\langle v_k,v \rangle | \ge t_k$ for every *k*. Here we present a streamlined version of Ball’s original proof.

A *plank* in a real finite-dimensional vector space is the region bounded by two parallel hyperplanes. For general Banach spaces, however, planks are defined with the aid of linear functionals: they are sets of the form $\{x\in X: |\phi (x)-m|\le w\}$, where *m* is a scalar, *w* is a non-negative number, and $\phi$ is a linear functional in $X^*$. In addition, if $\phi \in X^*$ has norm 1, the number *w* is called the *half-width* of the plank. Using this terminology, Ball’s affine plank theorem [2] states the following:

## Theorem 1

If the unit ball of a Banach space *X* is covered by a (countable) collection of planks in *X*, then the sum of the half-widths of these planks is at least 1.

For Hilbert spaces, the statement was proven almost 40 years earlier by Bang [4] in his remarkable solution to the famous plank problem of Tarski from the 1930s. As it stands, the plank theorem is sharp: any ball of a Banach space may be covered by a collection of non-overlapping parallel planks whose half-widths add up to 1. However, if one considers only planks which are symmetric about the origin, the condition of the theorem may be improved depending on the geometry of the space *X*. For example, for complex Hilbert spaces, the complex plank theorem of Ball [3] provides the optimal condition: the sum of the squares of the half-widths of the planks should be at least 1.

## Theorem 2

(Ball’s complex plank theorem)  Let $v_1,\dots ,v_n$ be unit vectors in ${\mathbb {C}}^d$, and $t_1,\dots ,t_n$, non-negative numbers satisfying $\sum _{k=1}^nt_k^2=1$. Then there exists a unit vector *v* in ${\mathbb {C}}^d$ for which $|\langle v_k,v \rangle | \ge t_k$ for every *k*.

Here $\langle u,v \rangle =\sum _{j=1}^du_j{\bar{v}}_j$ is the standard Hermitian inner product of $u,v\in {\mathbb {C}}^d$. For real Hilbert spaces, perhaps the most natural way to state the optimal condition is in the form of Fejes Tóth’s zone conjecture. A zone of spherical width *w* is the intersection of the sphere ${\mathbb {S}}^{d-1}$ and an origin-symmetric plank of half-width $\sin (w/2)$. In 1973, Fejes Tóth [5] conjectured that if *n* zones of equal angular width cover the sphere then their angular width is at least $\pi /n$. This conjecture was recently resolved by Jiang and Polyanskii [8] whose proof is based on the ideas of Bang [4] and Goodman and Goodman [7]. In [9] the author of the present note found a completely different proof inspired by Ball’s solution to the complex plank problem and Ambrus’s solution [1] of the strong polarization problem in the plane, which also follows Ball’s approach in [3]. Recently, Zhao [10] simplified the author’s proof of the zone conjecture by eliminating the reformulation of the problem in terms of Gram matrices. In this note, we simplify Ball’s original proof of Theorem 2 in a similar way.

A couple of months after this note was made public, Glazyrin et al. [6] noted that the proof by Zhao in [10] (in the real case) and the proof of this note (in the complex case) give some general information on zeros of real and complex polynomials restricted to the unit sphere. In particular, this observation allowed them to solved a generalization of Fejes Tóth’s problem for spherical segments (conjectured in [8]).

We begin with the following lemma that provides a simple characterization result of local extrema of functions defined on the unit sphere which are products of powers of the modulus of special linear factors. Throughout this note, we often use the natural identification ${\mathbb {C}}\cong {\mathbb {R}}^2$ where $z = x + iy$.

## Lemma 3

Let $v_1,\dots ,v_n$ be unit vectors in ${\mathbb {C}}^d$ and $t_1,\dots ,t_n$, positive reals satisfying $\sum _{k=1}^nt_k^2 = 1$. If *u* maximizes $\prod _{k = 1}^n|\langle v_k,u \rangle |^{t_k^2}$ among unit vectors, then

1 $$\begin{aligned} u = \sum _{k = 1}^n\frac{t_k^2}{\langle v_k ,u\rangle }v_k. \end{aligned}$$

## Proof

First observe that $|\langle v_k, u \rangle |>0$ for all *k* by the choice of *u*. Let $F:{\mathbb {C}}^d\rightarrow {\mathbb {R}}$ be the function defined by $F(v) = \prod _{k = 1}^n|\langle v,v_k \rangle |^{2t_k^2}$ for all $v\in {\mathbb {C}}^d$. The vector *u* is a solution to the problem of maximizing *F*(*u*) subject to the constraint $\Vert u\Vert ^2=1$, $u\in {\mathbb {C}}^d$. As a stationary point, $u \in {\mathbb {C}}^d$ satisfies the first order conditions and, as a consequence, the gradients of *F* and the constraint are proportional. This implies

2 $$\begin{aligned} \nabla _{{\bar{u}}} F(u) = \lambda \nabla _{{\bar{u}}} \Vert u\Vert ^2, \end{aligned}$$

for some $\lambda \in {\mathbb {R}}$, where $\nabla _{{\bar{u}}} = ({\partial }/{\partial {\bar{u}}_1}, \dots , {\partial }/{\partial {\bar{u}}_d})$ and

$$\begin{aligned} \frac{\partial }{\partial {\bar{u}}_j} = \frac{1}{2}\biggl (\frac{\partial }{\partial x_j} + i\frac{\partial }{\partial y_j}\biggr ) \end{aligned}$$

for all $j=1,\dots ,d$. Some simple properties of the operator $\nabla _{{\bar{u}}}$ are $\nabla _{{\bar{u}}} {\bar{u}} = {\mathbf {1}}$ where ${\mathbf {1}}$ is the vector point-wise equal to 1, $\nabla _{{{\bar{u}}}}u=0$, $\nabla _{{{\bar{u}}}}\langle v,u\rangle = v$, and $\nabla _{{\bar{u}}} \langle u, v\rangle =0$ for all $u,v \in {\mathbb {C}}^d$. It is also easy to check that $\nabla _{{\bar{u}}}\Vert u\Vert ^2 = \nabla _{{\bar{u}}} \langle u,u \rangle = u$, and $\nabla _{{\bar{u}}} |\langle v,u \rangle |^2 = \overline{\langle v,u \rangle }v$, for $v\in {\mathbb {C}}^d$. Thus,

3 $$\begin{aligned} \nabla _{{\bar{u}}} F(u) = \sum _{k = 1}^n\frac{\!\!F(u)t_k^2\,\,\,}{|\langle v_k , u \rangle |^{2t_k^2}} |\langle v_k , u \rangle |^{2t_k^2-2}\,\overline{\langle v_k,u \rangle }v_k= F(u)\sum _{k = 1}^n\frac{t_k^2}{\langle v_k , u \rangle }v_k. \end{aligned}$$

Therefore, (2) and (3) yield

4 $$\begin{aligned} F(u)\sum _{k = 1}^n\frac{t_k^2}{\langle v_k , u \rangle }v_k = \lambda u. \end{aligned}$$

Finally, taking inner product with *u* in both sides of (4) shows that $\lambda = F(u)$. $\square$

By compactness of the unit sphere in ${\mathbb {C}}^d$, Theorem 2 follows from the following theorem.

## Theorem 4

Let $v_1,\dots ,v_n$ be unit vectors in ${\mathbb {C}}^d$ and $t_1,\dots ,t_n$, positive reals satisfying $\sum _{k=1}^nt_k^2 = 1$. If *u* maximizes $\prod _{k=1}^n|\langle v_k ,u \rangle |^{t_k^2}$ among unit vectors, then $|\langle v_k ,u \rangle |\ge t_k$ for every *k*.

## Proof

Suppose for a contradiction that $|\langle v_1 ,u \rangle |<t_1$ (note that $|\langle v_k ,u \rangle |>0$ for every *k* due to the choice of *u*). We will examine the values of the product on vectors of the form $u_z = z v_1 + u$ for different complex numbers $z\in {\mathbb {C}}$ such that $\Vert u_z\Vert = 1$ (i.e., $u_z$ belongs to the complex unit sphere). Note that

$$\begin{aligned} \Vert u_z\Vert ^2 = |z|^2 +2{\mathfrak {R}}(z \langle v_1 , u \rangle ) + 1,\qquad z\in {\mathbb {C}}. \end{aligned}$$

Thus, $u_z$ belongs to the unit sphere if and only if $|z + \langle u ,v_1 \rangle | =|\langle v_1 , u \rangle |$, i.e., *z* belongs to the circle of radius $|\langle v_1 , u \rangle |$ centered at $-\langle u ,v_1 \rangle$. Let us call this circle *C*. The first factor of the product is also constant on *C* with value $|\langle v_1 ,u \rangle |$, since

$$\begin{aligned} |\langle u_z ,v_1 \rangle | = | z + \langle u ,v_1 \rangle | = |\langle v_1 ,u \rangle |, \qquad z \in C. \end{aligned}$$

Let $f:{\mathbb {C}}\rightarrow {\mathbb {R}}$ be the function defined by

$$\begin{aligned} f(z)=\prod _{k=2}^n\,\biggl |\frac{\langle v_k,u_z\rangle }{\langle v_k,u\rangle }\biggr |^{2t_k^2}=\prod _{k=0}^n\,\biggl |\frac{\langle v_k,v_1\rangle }{\langle v_k,u\rangle }{\bar{z}}+1\biggr |^{2t_k^2},\qquad z \in {\mathbb {C}}. \end{aligned}$$

Clearly, the maximum of *f*(*z*) on the circle *C* is $f(0)=1$ since $u_0 = u$ and *u* is a global maximizer of $\prod _{k=1}^n|\langle v_k,u\rangle |^{t_k^2}$ on the sphere. On the other hand,

$$\begin{aligned} \frac{\partial }{\partial {\bar{z}}}\biggl |\frac{\langle v_k ,v_1 \rangle }{\langle v_k ,u \rangle }{\bar{z}} + 1\biggr |^{2}&=\frac{\partial }{\partial {\bar{z}}}\biggl (\frac{\langle v_1 ,v_k \rangle }{\langle u ,v_k \rangle }z + 1\biggr )\biggl (\frac{\langle v_k ,v_1 \rangle }{\langle v_k ,u \rangle }{\bar{z}} + 1\biggr )\\&= \biggl (\frac{\langle v_1 ,v_k \rangle }{\langle u ,v_k \rangle }z + 1\biggr )\frac{\langle v_k ,v_1 \rangle }{\langle v_k ,u \rangle }, \end{aligned}$$

for all *k* where ${\partial }/{\partial {\bar{z}}}=({\partial }/{\partial x} + i{\partial }/{\partial y})/2$ (here we used basic properties of ${\partial }/{\partial {\bar{z}}}$ such as ${\partial z}/{\partial {\bar{z}}}= 0$ and ${\partial {\bar{z}}}/{\partial {\bar{z}}} = 1$). Thus,

$$\begin{aligned} \frac{\partial }{\partial {\bar{z}}}\bigg |_0\biggl |\frac{\langle v_k ,v_1 \rangle }{\langle v_k ,u \rangle }{\bar{z}} + 1\biggr |^{2} = \frac{\langle v_k ,v_1 \rangle }{\langle v_k ,u \rangle }. \end{aligned}$$

Therefore, by (1),

$$\begin{aligned} \frac{\partial f}{\partial {\bar{z}}}\bigg |_0&= \sum _{k = 2}^n t_k^2 \frac{\langle v_k ,v_1 \rangle }{\langle v_k ,u \rangle } = \left\langle \,\sum _{k = 2}^n \frac{t_k^2}{\langle v_k ,v_1 \rangle }v_k ,u \right\rangle \\&= \biggl \langle u-\frac{t_1^2}{\langle v_1,u \rangle }v_1, v_1 \biggr \rangle = \biggl (1 - \frac{t_1^2}{|\langle v_1 , u \rangle |^2}\biggr )\langle u , v_1 \rangle . \end{aligned}$$

Now we compute the real directional derivative of *f* in the direction of $w=-\langle u,v_1 \rangle$. We denote by $\nabla _{{\mathbb {R}}^2}$ the real gradient and by $\langle \,{\cdot }\,,\,{\cdot }\,\rangle _{{\mathbb {R}}^2}$ the standard inner product in ${\mathbb {R}}^2$. First note that, since *f* is a real valued function, $\nabla _{{\mathbb {R}}^2}f = 2{\partial f}/{\partial {\bar{z}}}$ where $z = x+ iy$. Hence,

$$\begin{aligned} \nabla _{{\mathbb {R}}^2}f(0) = -2\biggl (1 - \frac{t_1^2}{|w|^2}\biggr )w, \end{aligned}$$

and thus

$$\begin{aligned} \langle \nabla _{{\mathbb {R}}^2}f(0), w\rangle _{{\mathbb {R}}^2} = \biggl \langle -2\biggl (1 - \frac{t_1^2}{|w|^2}\biggr )w,w\biggr \rangle _{{\mathbb {R}}^2} = -2\biggl (1 - \frac{t_1^2}{|w|^2}\biggr ) |w|^2 . \end{aligned}$$

By assumption, note that $1 - t_1^2/|w|^2 < 0$. Therefore, by moving along the direction $w=-\langle u,v_1 \rangle$ from 0 into the open disc enclosed by the circle *C*, we find a complex number *z* in the open disc with $f(z) > 1$. Note that the logarithm of *f* is subharmonic; thus, by the maximum principle, this contradicts the choice of *u*. $\square$
